# Mo-Doped LSCF as a Novel Coke-Resistant Anode for Biofuel-Fed SOFC

**DOI:** 10.3390/ma17040869

**Published:** 2024-02-13

**Authors:** Kimia Y. Javan, Massimiliano Lo Faro, Sebastian Vecino-Mantilla, Vincenzo M. Sglavo

**Affiliations:** 1 Department of Industrial Engineering, University of Trento, Via Sommarive 9, 38123 Trento, Italy; vincenzo.sglavo@unitn.it; 2 Institute for Advanced Energy Technologies (ITAE) of the National Research Council of Italy (CNR), Via Salita S. Lucia sopra Contesse 5, 98126 Messina, Italy; lofaro@itae.cnr.it (M.L.F.); vecino@itae.cnr.it (S.V.-M.); 3 National Interuniversity Consortium of Materials Science and Technology (INSTM), University of Trento Research Unit (UdR Trento), Via G. Giusti 9, 50121 Firenze, Italy

**Keywords:** IT-SOFC, molybdenum, La_0.6_Sr_0.4_Co_0.2_Fe_0.8_O_3−δ_, coke-tolerant, biofuel, anode

## Abstract

Climate change and damage to the environment, as well as the limitations of fossil fuels, have pushed governments to explore infinite renewable energy options such as biofuels. Solid Oxide Fuel Cell (SOFC) is a sustainable energy device that transforms biofuels into power and heat. It is now being researched to function at intermediate temperatures (600–700 °C) in order to prevent material deterioration and improve system life span. However, one of the major disadvantages of reducing the temperature is that carbon deposition impairs the electrochemical performance of the cell with a Ni-YSZ traditional anode. Here, molybdenum was doped into La_0.6_Sr_0.4_Co_0.2_Fe_0.8_O_3−δ_ (LSCFMo) as an innovative anode material with higher coke resistance and better phase stability under reducing conditions. X-ray diffraction (XRD) analysis showed increasing phase stability by increasing the Mo dopant. Electrochemical measurements proved that the LSCFMo anode is an active catalyst towards the methanol oxidation even at low temperatures as 600 °C, with an open circuit voltage (OCV) of 0.55 V, while GDC10 (Ga_0.9_Ce_0.1_O_1.95_) is used as the electrolyte. As an insightful result, no trace of any carbon deposition was found on the anode side after the tests. The combination of phase composition, morphological, and electrochemical studies demonstrate that LSCFMo is a suitable anode material for SOFCs fed by biofuels.

## 1. Introduction

Climate change and the growing demand for renewable energy sources have led to research on advanced power systems like solid oxide fuel cells (SOFC) which can reach an efficiency of up to 60% [1]. Biogas and methane have emerged as potential SOFC fuels due to their abundance, low price, and limited contribution to annual carbon dioxide emissions [2]. Methane internal reforming into hydrogen is usually carried out by traditional nickel-based anodes in cells operating at high temperatures (800–1000 °C). Nevertheless, the main issue at high temperatures is the thermal cracking of methane and the subsequent carbon deposition, due to the strong catalytic activity of nickel [3], which can lead to a rapid degradation of the electrochemical cell in terms of obstruction of pores for gas diffusion, expansion of anode support, and consequently breaking of the cell [4]. Therefore, the performance starts to deteriorate, usually before reaching a lifetime of 40,000 h which strongly calls into question the viability of commercializing biogas-fed SOFC.

To address such issues, research has prompted the development of SOFC operating at intermediate temperatures (IT-SOFC), down to 500–700 °C, thus offering many advantages such as faster start-up and shutdown, slower corrosion of interconnect or sealant materials, improved cell durability, and lower costs [5]. All these positive aspects are accounted for by reduced coarsening of catalysts [6] and lower residual stresses in components of different thermal expansion coefficients (TEC) [7]. The downside, though, is the lower specific ionic conductivity of the electrolyte [8] and higher electrode overpotentials [9] which limit the feed flexibility at low temperatures. Carbon deposition can also take place as a consequence of Boudouard or reverse water–gas reactions [10]. Sluggish electrocatalytic activity at T < 750 °C leads to an incomplete reforming reaction which ends with reducing CO to solid carbon [11,12].

There is a wealth of literature on the replacement of nickel-based zirconia with doped-perovskites that benefit from higher coke resistance and better electrocatalytic activity at low temperatures while maintaining the electronic-ionic conductivity (MIEC properties) [13]. La_0.6_Sr_0.4_Co_0.2_Fe_0.8_O_3−δ_ (LSCF) represents the most investigated perovskite used as electrode material, it being also doped by nickel to induce better catalytic activities towards biofuels oxidation [14,15]. Nevertheless, their weak structural stability under reducing conditions has still hindered the practical application of perovskites [16]. Few studies have shown the possibility of applying LSCF as an anode while it can decompose to Ruddlesden–Popper (R.P.) oxides and CoFe nanoparticles under low oxygen partial pressures. In case LSCF is doped, the dopants are exsolved together with nanoparticles on the surface and such exsolved alloys provide the structure with improved catalytic activity, better thermal stability, and higher coke resistance [17]. Although higher-order R.P. oxides exhibit enhanced electroconductivity [18], first-order newly generated R.P. oxides after LSCF reduction, such as (La_0.38_Sr_0.62_)_2_FeO_4_ and La_2_CoO_4_, have been shown to have good but lower electroconductivity than the parent perovskite. Park et al. [19] and Chung et al. [20] attributed the explanation to fewer B-O-B bridges in first-order R.P. oxides, which often serve as the electron channel.

B-site doping in LSCF with molybdenum as a high valence acceptor dopant can improve redox stability to a great extent. Previous studies proved that by doping Mo into Ba_0.5_Sr_0.5_Co_1−x_Fe_x_O_3−d_ [21,22], and La_0.5_Sr_0.5_FeO_3−δ_ [23] the reduction stability increased remarkedly. Meanwhile, the electrochemical performances were increased. It is noticeable that Mo is an element with a higher proportion of unoccupied 3D-orbitals which makes it an active electrocatalyst. Li et al. [24] confirmed better catalytic activity and performance increasing after doping Mo into La_0.3_Sr_0.7_TiO_3−d_. Wang et al. [25] included Mo into La_0.6_Sr_0.4_FeO_3−δ_ and achieved higher electrocatalytic activity at the surface towards CO_2_ electrolysis, due to Fe–O–Mo pairs which can store higher oxygen ions. In another work, Liu et al. [26] found better electrocatalytic activity toward oxygen reduction reaction by doping Mo into LSCF cathodes. Another insightful advantage of doping Mo is the significant boosting in carbon tolerance of the perovskite. He et al. [27] highlighted the remarkable carbon tolerance of Sr_2_TiNi_0.5_Mo_0.5_O_6_. In 2012, Li et al. [28] postulated that the high carbon tolerance of Mo-included oxides like Sr_2_Fe_1.5_Mo_0.5_O_6_ is due to two main reasons. First, their higher structural stability prevents further metal cluster formation which hampers enough surface provide for carbon deposition. Secondly, Mo doping in an oxide structure is associated with more oxygen vacancies which are formed just around the active B-site cations like Mo^6+^. Therefore, the instant formation and consumption of activated carbon species increases the tolerance of the anode towards carbon adsorption. These are other positive points in Mo doping like increasing electronic conductivity [29] or reducing the thermal expansion coefficient [30]. 

Despite the sporadically reported benefits of Mo doping into different perovskite structures, and even in LSCF, no studies are available on possible applications of Mo-doped LSCF as anode material in SOFC. It is crucial to note that the advantages of Mo doping are also dependent on the cell design, materials, and thickness of other cell components, electrode microstructure, and even the crystal environment around Mo ions.

The present work aims at developing an IT-SOFC directly fed by biofuels. An innovative anode material is adopted, specifically B-site doped LSCF with Mo, with the goal of reducing the carbon deposition on the anode side as much as possible. The powder is synthesized by a modified auto-combustion method and its phase stability under reduction is determined. Subsequently, the cell is fabricated by colloidal processes and co-sintering. The cell’s electrocatalytic activity and performance are assessed using dry methanol as a biofuel. 

## 2. Materials and Methods

### 2.1. Powder Synthesis

La_0.6_Sr_0.4_(Co_0.2_Fe_0.8_)_1−x_Mo_x_ O_3−δ_ (LSCF (x = 0), LSCFMo5 (x = 0.05), and LSCFMo10 (x = 0.1)) powders were synthesized using an auto-combustion method. The stoichiometric amounts of salts (CARLO ERBA RPE Reagents SRL): lanthanum nitrate hexahydrate (La(NO_3_)_3_.6H_2_O, lot. V8F488118G), cobalt nitrate hexahydrate (Co(NO_3_)_2_.6H_2_O, lot. V9C134160A), ferric nitrate nonahydrate (Fe(NO_3_)_3_.9H_2_O, lot. V8D575309I), ammonium heptamolybdate tetrahydrate ((NH_4_)_6_Mo_7_O_24_.H_2_O, lot. V9I123200B), and strontium nitrate ((Sr(NO_3_)_2_, Acros Organics, lot. A0419899) were dissolved and mixed in distilled water. Enough ammonium nitrate ((NH_4_)NO_3_, lot. 0888C100) was added as an oxidant aid. The fuel components, ethylenediaminetetraacetic acid (EDTA, lot. 0387I100), citric acid (CH_3_COOH, J.T.Baker, lot. 0110110019), and polyethylene glycol (PEG 20000, Alfa Aesar GmbH, lot. I6622A), were mixed separately in a 1:2:3 ratio to the total metal cations. The amount of added ammonium hydroxide (NH_4_(OH)) was adjusted to reach a solution with 6 < pH < 8. This mix was then merged with the first one and the obtained solution was stirred at 80 °C until a reddish-brown gel structure was achieved. The gel was heated at 250 °C and a rapid auto-combustion reaction took place resulting in a black ash powder, which was further calcined at 1000 °C for 4 h.

### 2.2. Half-Cell Preparation

Gadolinium-doped ceria (GDC) electrolyte layer was prepared by tape casting. For the slurry preparation, water as a solvent and DARVAN 821-A as a dispersant (1.2 wt%) were mixed and GDC commercial powder (Ga_0.9_Ce_0.1_O_1.95_—GDC10, SSA = 10–15 m^2^/g) was added to the mixture until achieving 75% of solid loading. The mixture was ball-milled for 3 h in a Turbula (T2F, WA BACHOFEN AG, CH) mixer and then Duramax B1000 was added as a binder agent. The lanthanum strontium ferrite—LSF cathode layer was prepared by a similar tape casting procedure, using LSF commercial powder (La_0.8_Sr_0.2_FeO_3−δ_—LSF20, SSA = 9.7 m^2^/g, NexTech Materials). To achieve the necessary porous structure for the cathode, the solid loading was optimized at 47%, and corn starch (10 wt%) was added as a pore former together with the LSF powder. PEG4000 was added as the plasticizer together with Duramax B1000 binder at an appropriate ratio (1.0 wt%) in LSF slurry. The ratio between the plasticizer and binder was finely tuned to achieve a green tape with enough flexibility and, at the same time, good mechanical stability. The mixtures were mechanically stirred for 2 h at 150 rpm. Afterward, the suspensions were cast on a polyethylene terephthalate (PET) tape carrier using a doctor blade apparatus with a gap of 80 and 900 μm for GDC and LSF, respectively. The relative humidity in the chamber was controlled at 70%. The cast tapes were dried after 72 h. Then, GDC and LSF green tapes were laminated together by a Carver hydraulic thermopress (20 MPa/75 °C/10 min, FRED S. CARVER INC., Menomonee Falls, WI, USA) to produce the GDC|LSF half-cell.

### 2.3. SOFC Fabrication

To fabricate button SOFCs, LSCFMo anode ink was screen-printed onto the GDC side of the half-cell previously realized. To prepare the screen-printing ink, a vehicle of 94 wt% α-terpineol as a solvent and 6 wt% ethyl cellulose (EC, Sigma-Aldrich Co., St. Louis, MO, USA, lot. MKBV7094V) as a binder was prepared. The synthesized LSCFMo powder was added to the vehicle to reach 27% solid loading. The mixture was mechanically stirred for 1 h and then Triton-x100 (Sigma-Aldrich Co., St. Louis, MO, USA, lot. SLBB0703V) was added up to 7 wt% of the powder, for better thixotropic behavior after printing and also to prevent possible cracks after sintering [31]. The ink was screen printed onto the GDC layer through a circle stencil with 15 mm diameter and 250 mesh. Considering the sintering shrinkage, disks with a diameter of 24 mm with printed anode at the center were produced.

The green button cells were co-sintered with a two-stage sintering program, the first step at 600 °C for 1 h (heating ramp = 0.5 °C/min) and the second one at 1225 °C for 3 h (heating ramp = 2 °C/min).

### 2.4. Microstructure and Anodic Stability of LSCFMo

Powder particle size and morphology were assessed by field-emission scanning microscopy (FE-SEM) using ZEISS^®®^ Supra 40 microscope (Carl Zeiss Microscopy GmbH, Jena, Germany), and the crystallographic structures were characterized by XRD. The elemental analysis of the synthesized powder was detected by energy dispersive X-ray spectroscopy (EDS) using Jeol IT300 SEM equipped with Xflash 630 M detector (Bruker Quantax) (JEOL Ltd., Tokyo, Japan).

The microstructure of the anode and cathode surfaces and the cross-section morphology of the cells were observed by SEM.

Subsequent studies investigated the microstructure and mechanical stability of LSCFMo powder under reducing conditions. For these studies, green tapes prepared from LSCFMo were sintered at 1225 °C for 3 h. Then, they were subjected to 5% H_2_/Ar gas flow as a reducing agent at 900 °C (heating rate = 5 °C/min) for 10 h. The phase stability of the tapes was assessed by XRD and the mechanical stability was tested manually. The bulk density of sintered and reduced tapes was evaluated by SEM analysis with the aid of image analysis software (ImageJ 1.54f). The surface porosity of the tape was assessed by proper threshold adjustment of the image and calculating the pixel ratio with respect to the total pixels in the image area.

### 2.5. Electrochemical Characterization

To carry out the electrochemical and performance measurements, each produced cell was mounted on an alumina tube using a ceramic paste (Aremco) for sealing gas fuel on the anodic side. The cathode side was exposed to static airflow. Gold wires and paste were applied to both electrodes serving as electrical current collectors. SOFC open circuit voltage (OCV) measurements were carried out at 600 °C at a total flow rate F = 50 cm^3^/min in three stages. A pre-treatment of the LSCFMo anode was performed using an inert He (50 cm^3^/min) gas flow for the time necessary to ensure that the OCV was stable and to clean any organic residue derived from sealing paste or gold decomposition off the anodic tube. Then, the cells were fed first by dry biogas (30 cm^3^/min CH_4_ + 20 cm^3^/min CO_2_) and then by pure methane (50 cm^3^/min). The cells were fed by liquid methanol using a syringe pump and He as a carrier (0.5 cm^3^/h CH_3_OH + 25 cm^3^/min He) at 600 °C, to understand the mechanism and compare the catalytic activity towards the fuel oxidation. To examine the effect of He carrier on methanol oxidation, two amounts of He flow rate (10 and 50 cm^3^/min) were tested. Short-term durability tests were carried out at 600 °C with methanol and at 700 °C with both H_2_ (50 cm^3^/min) and methanol as fuels, under galvanostatic conditions. Electrochemical impedance spectroscopy (EIS) measurements and polarization curves (current-potential I-V curves) were examined. Carbon presence on the anode side was evaluated optically.

## 3. Results and Discussion

### 3.1. Powder Characterization

**Figure 1:**
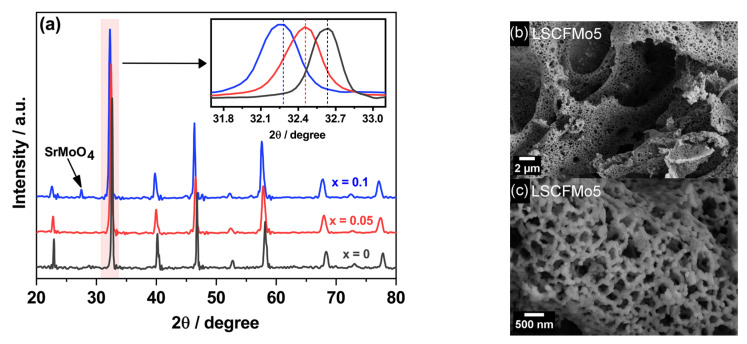
(**a**) XRD spectra for as-synthesized LSCFMo_x_ (x = 0, 0.05, 0.1) powders; (**b**,**c**) FE-SEM images of LSCFMo5.

LSCFMo_x_ (x = 0, 0.05, 0.1) powder was synthesized successfully by the modified auto-combustion method, including PEG 20000 in addition to EDTA and citric acid as fuel aids. EDTA and citric acid can chelate to metal cations very strongly which subsequently increases the activation energy for metal cations reduction which is accompanied by a slow combustion process. This hampers the desired perovskite structure formation [32]. The polymer PEG 20000 was added in the current investigation to improve the combustion intensity, which led to pure phase formation. This is because PEG and other polymeric fuels form complexes with non-chelated cations not by covalent bonding but rather by trapping them inside the shell of a kind of micelle [33]. Due to weak interactions with polymeric chains, the metal cations that are chelated in this manner can be reduced more quickly. This compensates for citric acid’s less intense combustion and increases the final yield [34,35]. The XRD pattern in Figure 1a indicates that the LSCFMo rhombohedral phase is synthesized successfully. The peaks shift to lower angles by increasing the Mo dopant at the B-site. This is both due to the larger ionic radii of Mo^6+^ (0.59 Ǻ) with respect to Fe^4+^ (0.58 Ǻ) and Co^4+^ (0.53 Ǻ) and because, after Mo doping, some Fe^4+^ and Co^4+^ are reduced to Fe^3+^ and Co^3+^ to fulfill electroneutrality in the structure [36]. The peak at about 27.5° is related to 5 wt% SrMoO_4_ that was detected as an impurity in LSCFMo10 powder. The real Mo dopant amount was found to be around 8% instead of 10%, using peak analysis Maud software. This indicates that Mo could not be doped completely into B-site and is assumed to be the result of pH variations during powder synthesis. At lower and higher pH, cations’ oxidation state changes and this hampers their participation in the formation of the desired crystal structure [33]. SEM pictures of LSCFMo5 are shown in Figure 1b,c which reveals a very porous structure with particles of the size 50–200 nm. The same morphology was observed for LSCF and LSCFMo10.

**Figure 2:**
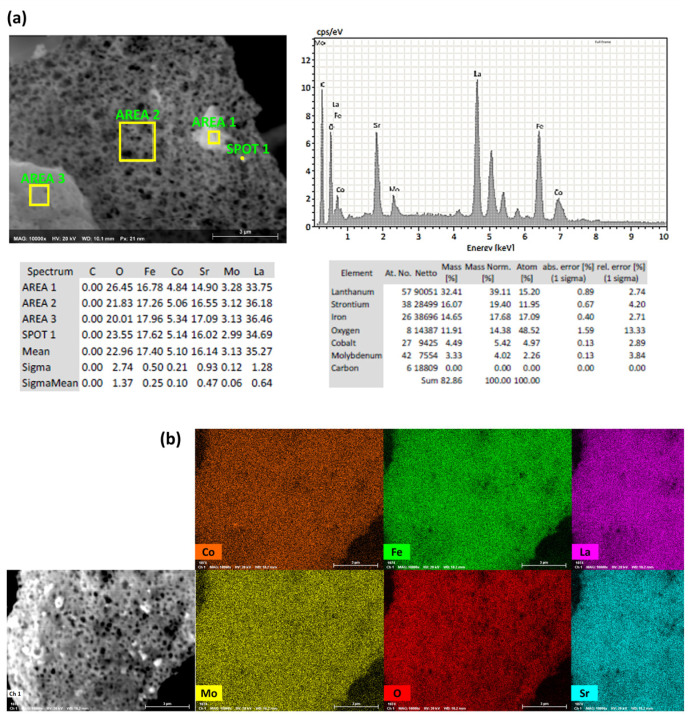
EDS analysis including (**a**) compositional profile and (**b**) elemental distribution of the synthesized LSCFMo powder.

Elemental analysis of the synthesized LSCFMo is shown in Figure 2. As proven by the compositional profile of the particles in different spots in Figure 2a, the powder composition is homogeneous. Elemental distribution of LSCFMo is shown in Figure 2b. The elements La, Sr, Co, Fe, and Mo are distributed homogeneously in the powder and no obvious secondary phase or segregation after synthesis and calcination is observed.

### 3.2. Cell Design and Microstructure

**Figure 3:**
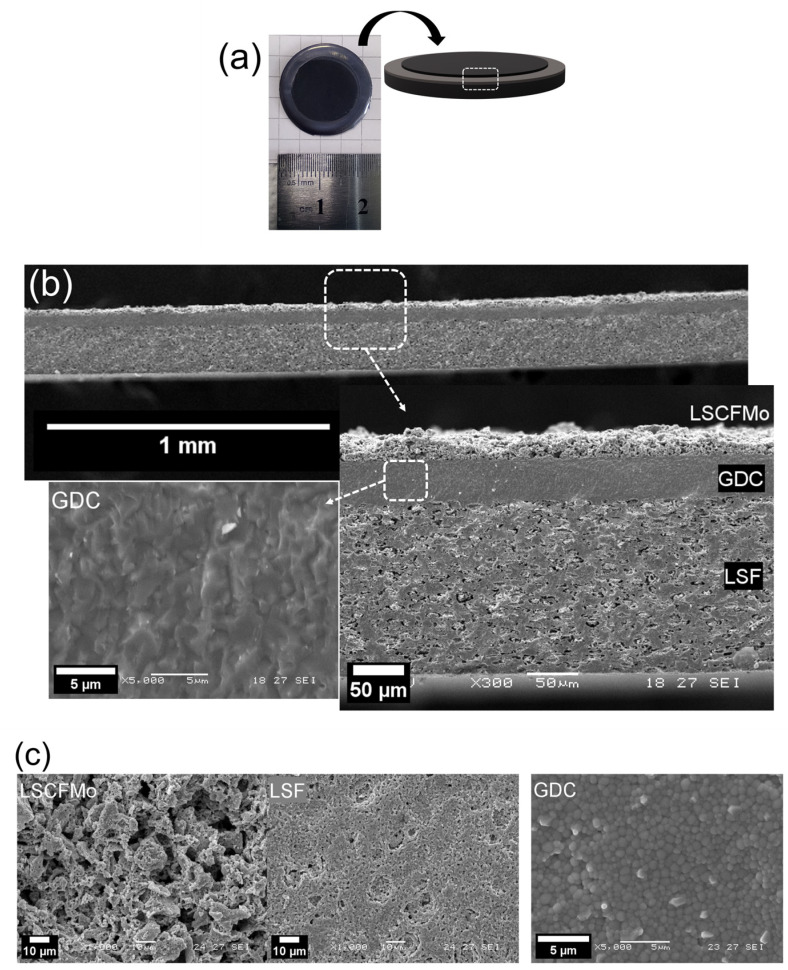
(**a**) Photograph and 3D schematic of a co-sintered button-cell, SEM images of (**b**) cell cross-section, and (**c**) anode, cathode, and electrolyte surface after co-sintering (this latter corresponds to non-coated regions on the border of the cell).

After co-sintering, integrated and flattened button-SOFC with good mechanical properties were obtained. Mo5 and Mo10 will now refer to cells with anodes made of LSCFMo5 and LSCFMo10, respectively. Figure 3 shows the final cell design and structure. The cross-section microstructure of the cells after co-sintering is shown in Figure 3a. The cell consists of an LSCFMo porous anode, a thick GDC electrolyte, and an LSF cathode. The thickness of the LSF cell’s support layer is 170 µm; the thickness of the GDC electrolyte is 40 µm. While LSF and GDC benefit from a uniform thickness as a result of the tape casting process, LSCFMo anode thickness varies in the range of 20–35 µm after repeated screen-printing. Both the anode and cathode are well-attached to the GDC electrolyte and no delamination is observed throughout the entire cell. Lu et al. [30] and Zhang et al. [36] found that Mo doping into Pr_0.6_Sr_0.4_(Fe_0.8_Ni_0.2_)_1−x_Mo_x_O_3−δ_ and Pr_0.4_Sr_0.6_(Co_0.2_Fe_0.8_)_1−x_Mo_x_O_3−δ_, respectively, decreased the TEC significantly as Mo dopant was increased. The presence of a high-valent element like Mo in the structure can suppress the reduction in B-site cations (Fe^4+^, Co^3+^, Ni^4+^) to their lower valence states (Fe^3+^, Co^2+^, Ni^3+^) and thus, the TEC is decreasing. As a result, the compatibility between electrode and electrolyte is increased, delaying cracking and failure in the cell during heating or redox cycles. The surface microstructure of LSCFMo, LSF, and GDC is shown in Figure 3b. After co-sintering, a total porosity of 36% for the anode and 15% for the cathode was determined, these being adequate for fuel and oxygen availability at triple phase boundaries (TPBs). A dense GDC layer is achieved with only some isolated and small pores. The cross-section SEM image of GDC shows almost merged grains, yet when the surface is examined, grain boundaries can be detected (Figure 3c).

### 3.3. Anodic Stability

**Figure 4:**
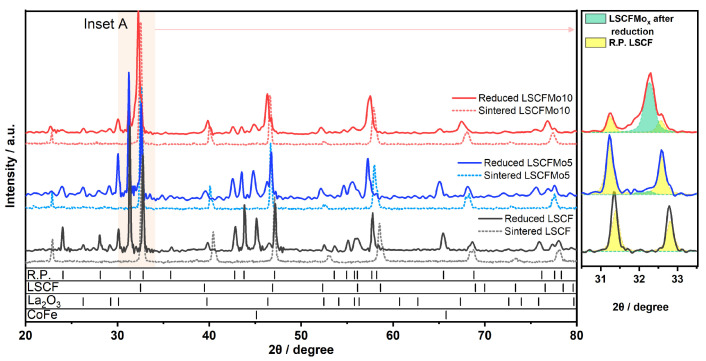
XRD pattern of LSCF, LSCFMo5, and LSCFMo10 after reduction in Ar/H_2_ at 900 °C for 10 h.

XRD analysis was used to investigate the influence of Mo^6+^ doping and partial replacement for Fe^4+^ on the phase and microstructural stability of LSCF, as shown in Figure 4. After the reduction in H_2_, LSCF was entirely dissolved into R.P. oxides and CoFe alloy nanoparticles. The phase stability was improved by increasing the Mo dopant concentration. LSCFMo5 dissolved into R.P. oxides and CoFe nanoparticles but it retained some of its perovskite rhombohedral structure. This is in contrast with respect to LSCFMo10, which demonstrated excellent phase stability after reduction and could be retained up to 60% with just a small quantity of newly produced R.P. oxides. The peak ratio between the residual perovskite (green in Figure 4) and the newly created R.P. oxides (yellow) after reduction is shown in the inset (A) of Figure 4. By increasing the Mo dopant, this ratio increases. It is thought that under low partial oxygen pressures, an ion with a large radius, such as Mo^6+^ in the B-site, can accommodate Sr^3+^ in the A-site, suppressing Sr activation and dissolution [37]. Furthermore, Mo^6+^ can moderate further reduction in Fe^3+^/Fe^2+^ into the metallic phase, thus preventing the collapse of the perovskite structure. This is because the redox bands of mixed-valent Mo^5+^/Mo^6+^ and Fe^3+^/Fe^2+^ couples overlap [38]. The main peak for LSCFMo is shifted before and after reduction thus pointing out a variation of the unit cell volume and parameters induced by the heat treatment but not corresponding to some kind of decomposition.

**Figure 5:**
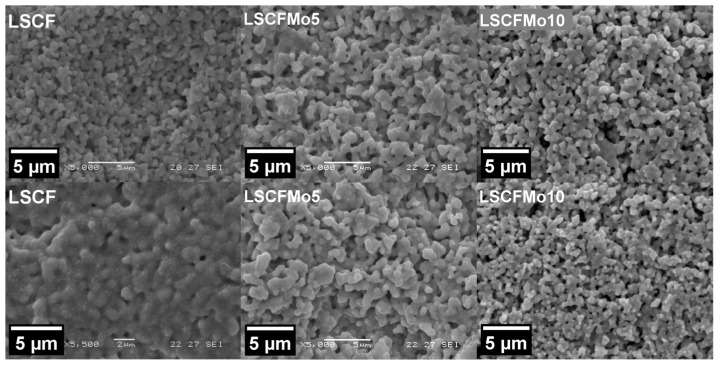
SEM images of the surface of sintered (top) and reduced (bottom) LSCF and LSCFMo tapes.

Figure 5 shows the SEM images taken from the surface of as-sintered and reduced LSCFMo tapes. Along with the results from XRD, the tape LSCFMo10 shows the most retained microstructure and porosity following reduction. The LSCFMo5 tape shows a larger grain size and higher (about 30%) bulk density after reduction. Due to the complete decomposition of LSCF after reduction and the larger cell volume of R.P. oxides with respect to LSCF, the bulk density is increased by about 70%.

**Figure 6:**
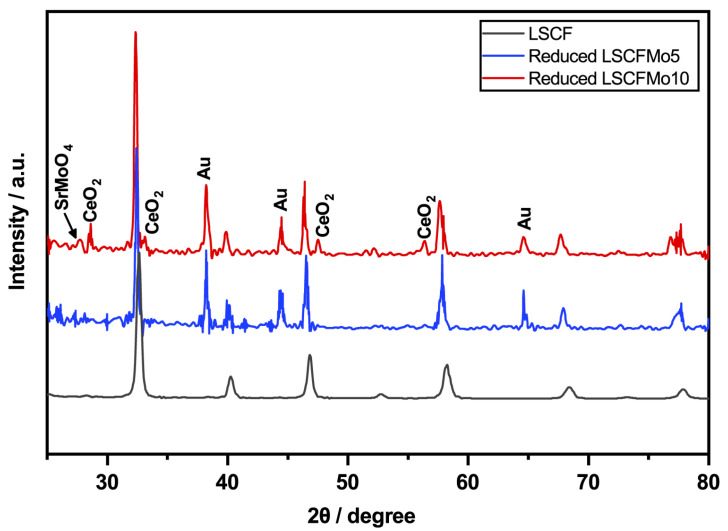
XRD pattern of LSCF, LSCFMo5, and LSCFMo10 after electrochemical tests and reduction in methanol at 600 °C.

Figure 6 shows the XRD patterns of LSCFMo after electrochemical tests. After the reduction in methanol, the phase stability is preserved for both LSCFMo5 and LSCFMo10 with no R.P. oxides formation. They are observed strong peaks of Au which are diffracted from the gold current collector paste. Some less intense peaks of ceria are observed in the LSCFMo10 pattern. There is the possibility that in some little spots, the screen printed anode layer is too thin or even, due to not full coverage in those spots, X-ray could reach the electrolyte.

### 3.4. Electrochemical Measurements

#### 3.4.1. OCV and Durability

**Figure 7:**
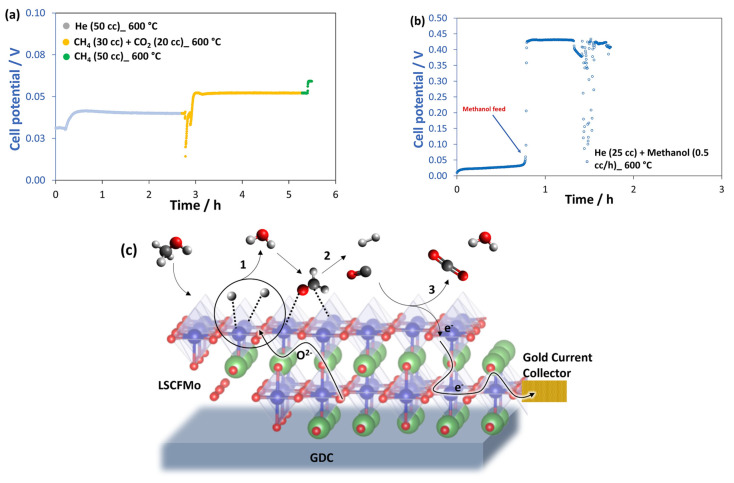
OCV measurements at 600 °C of the cells fed by (**a**) He, biogas, and pure methane and (**b**) He and methanol. (**c**) Suggested schematic of “shuttle mechanism” in methanol oxidation on Mo-doped LSCF.

Figure 7a shows OCV measurements. During the pre-treatment of the anode with He gas at 600 °C, a very low OCV (0.04 V) is recorded. When the cell is supplied with biogas (30 cm^3^/min CH_4_ + 20 cm^3^/min CO_2_), the potential increases to 0.05 V, which is still lower than the practical potential. The cells were then supplied with pure dry methane at the following stages. The potential is only marginally enhanced, reaching 0.06 V, as a result of the higher partial pressure of CH_4_. Despite the fact that the potential is steadily independent of fuel type, the low OCV shows that LSCFMo has extremely weak electrocatalytic activity and acts as an inert for direct methane oxidation. 

To investigate the best biofuel and catalytic mechanism for LSCFMo, the cell was fed methanol (0.5 cm^3^/h CH_3_OH + 25 cm^3^/min He) at 600 °C. Figure 7b shows the OCV measurements. Surprisingly, the cell potential quickly increases. In LSCFMo, which works with biofuels other than biogas and methane, the “shuttle mechanism” is the main mechanism. Lo Faro et al. [39] proposed the shuttle mechanism to occur when dry alcohol fuels are used with lanthanum ferrites. Lanthanum ferrites are not powerful enough as anodic catalysts to perform direct oxidation of biofuels, while methanol is oxidized through several subsequent processes. “Shuttle mechanism” begins with the dehydrogenation of methanol and then, the oxidation of CO and H_2_ residues (Figure 7c). It is suggested that the methanol dehydrogenation and cracking-free reactions are promoted by molybdenum dopant in LSCF, and the reactions are given by

(1) $${CH}_{3}OH\overset{LSCFMo}{\to }{-CH}_{2}O_{\left(ads\right)}+H_{2}\;Dehydrogenation$$

(2) $$H_{2}+O^{2-}\overset{LSCFMo}{\to }H_{2}O+{2e}^{-}\;Oxidation$$

(3) $${-CH}_{2}O_{\left(ads\right)}+H_{2}O\overset{LSCFMo}{\to }2H_{2}+{CO}_{2}$$

(4) $${CO}_{2}+H_{2}\overset{LSCFMo}{\to }CO+H_{2}O$$

(5) $$CO+O^{2-}\overset{LSCFMo}{\to }{CO}_{2}+{2e}^{-}\;Oxidation$$

Although LSCFMo shows good catalytic activity towards methanol oxidation, the potential is low, probably because GDC10 becomes partially electronically conductive. The overall voltage loss after 1.5 h, on the other hand, could be reduced by adjusting the fuel flow settings.

**Figure 8:**
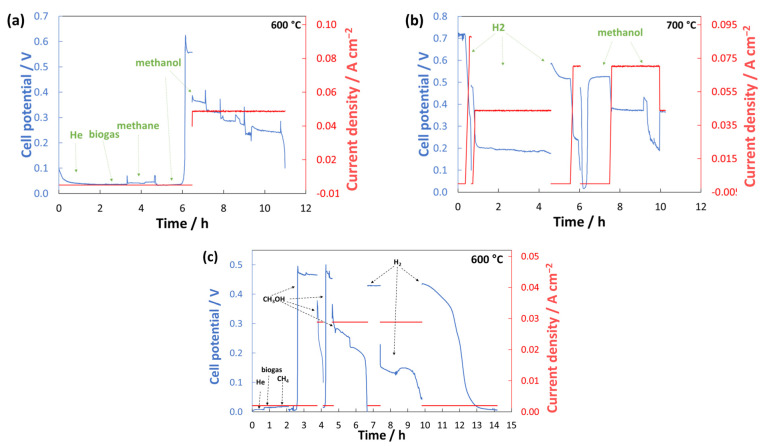
Short-term durability of cells (**a**) Mo5 at 600 °C, (**b**) Mo5 at 700 °C, and (**c**) Mo10 at 600 °C.

Figure 8 shows the short-time durability tests of the cells Mo5 and Mo10 while being fed by different fuels at 600 °C and 700 °C. In line with previous OCV studies, a very low potential is recorded at an open circuit current when the cells are fed by biogas and methane. The potential is boosted to 0.55 V after feeding the cell Mo5 with methanol at 600 °C, although the performance diminishes rapidly after 5 h. This value is just slightly lower than the average OCV for SOFC with GDC10 electrolyte (around 0.60 V [16]). One can see in Figure 8b that increasing the temperature to 700 °C does not make a big difference in OCV, although the performance degrades slower. The performance becomes stable when the cell is fed by hydrogen. With H_2_ at 700 °C, an OCV of 0.74 V is recorded, and the potential drops down to 0.20 V when the current is loaded. Nevertheless, the performance is stable for almost 4 h. Figure 8c shows that Mo10 behaves similarly to Mo5 at 600 °C. Feeding Mo10 with methanol, the achieved OCV is 0.47 V but with a rapid decline after loading current. Because He may trigger the removal of methanol fuel from the anode’s active sites, less He was injected into the flow. As a result, the performance deterioration rate is minimized. The deterioration rate may be improved by adjusting the flow rate and fuel ratio. After that, the cell was fed by pure H_2_ for about 7 h. Although the OCV is a bit lower than Mo5, it is assumed to be related to earlier defects developed in the cell Mo10 during the tests. Nevertheless, this OCV shows a certain reactivity of LSCFMo towards hydrogen and methanol oxidation although it is gradually lost.

#### 3.4.2. EIS

**Figure 9:**
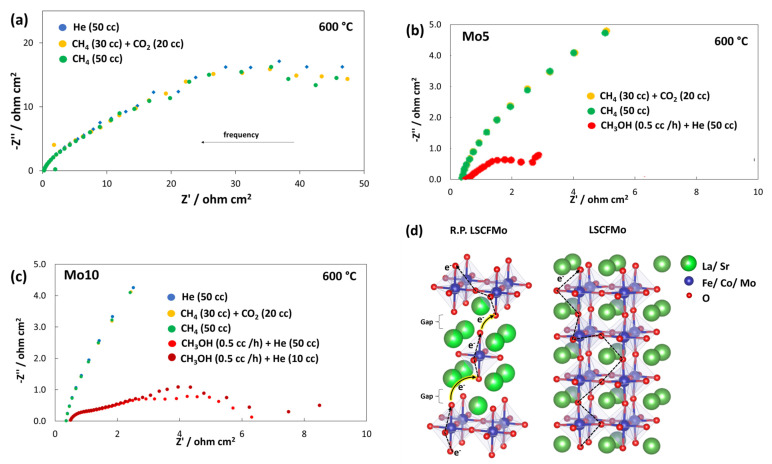
EIS plots of the cells at 600 °C when fed by (**a**) H_2_, biogas, and methane, (**b**) methanol in cell Mo5, and (**c**) methanol in cell Mo10. (**d**) Charge transfer in LSCFMo and its R.P. oxide.

Each EIS spectrum contains information concerning the series resistance (R_S_), which is primarily caused by the ionic resistance of the electrolyte, and RQ elements in series, which are ascribable to cell reaction resistances. Figure 9 shows the impedance spectra for different fuels at 600 °C. Figure 9a shows three RQ elements for both Mo5 and Mo10 cells when fed by biogas and methane. The large semicircles imply a significant kinetic barrier to direct methane oxidation. A smaller semicircle at high frequencies indicates the low activation control on the cathode side. It is porous enough and air can easily access TPBs. However, the larger semicircles at medium and low frequencies are taken as the poor electrocatalytic activity of LSCFMo towards the direct methane oxidation and related oxidation intermediates. Nevertheless, R_S_ is approximately 0.20 Ω cm^2^ and does not vary significantly with the type of fuel which is seen as a positive aspect.

Figure 9b,c show the EIS spectra from the cells Mo5 and Mo10, respectively, fed by methanol. The series resistance is as low as 0.14 Ω cm^2^ in Mo10 which indicates the good innovative configuration and design of the cell. At least two RQ elements are visible in both cells, with additional semicircles in Mo10. The first and second RQ are related to the dehydrogenation of methanol, and CO and H_2_ oxidations on the anode side, respectively. It was previously confirmed that LSCFMo10 preserves the perovskite structure under reducing conditions, while LSCFMo5 is more decomposed to R.P. oxides. Figure 9d shows LSCFMo and its R.P. oxide structure. Higher reduction stability in LSCFMo10 can protect the bond bridge Fe^3+^–O–Fe^4+^ in the structure which prevents further increase in the activation energy of electron transfer [19,40]. Whereas, in LSCFMo5, more activation energy is required to transfer the electron due to the gap between layers. As a result, the first semicircle which is affected by the activation control of anodic reactions is smaller in Mo10 than in Mo5. A similar result in enhancing charge transfer by Mo doping into lanthanum ferrites was proved by Hou et al. [40], who showed that higher reduction stability can also bring about an increase in electroconductivity of Mo-doped La_0.6_Sr_0.4_Fe_0.9_Ni_0.1_O_3−δ_, at a wide temperature range (500-–850 °C). The additional RQ elements in Mo10 are related to O_2_ reduction on the cathode side and the adsorption and dissociation of dry organic molecules which gets facilitated as the He carrier arises. The total polarization resistance is larger in cell Mo5 than in Mo10.

#### 3.4.3. I-V Curves

**Figure 10:**
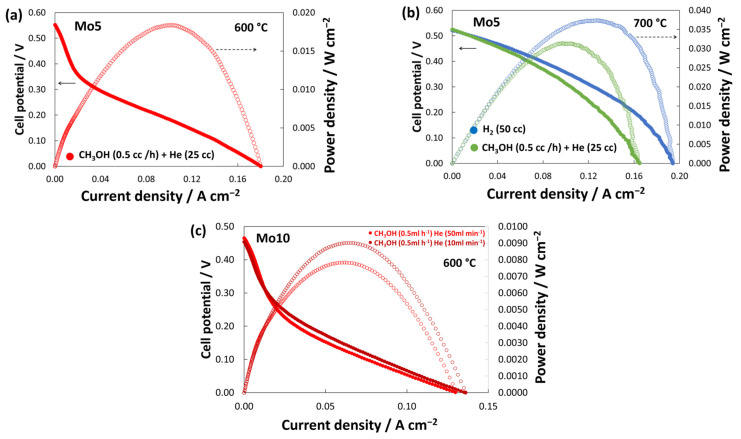
Polarization curves for the cells (**a**,**b**) Mo5 at 600 °C and 700 °C, and (**c**) Mo10 at 600 °C.

Figure 10 compares the polarization curves of the various tested cells. The fast drop in voltage, around 200 mV, indicates an increase in activation control in both cells towards methanol oxidation, due to overvoltage after loading the current. By increasing the operating temperature to 700 °C (Figure 10b), the activation control perfectly disappears and the maximum power density is almost doubled. This is because at a higher temperature, charge transfer is facilitated, and the electrochemical activity increases. However, due to low OCV, the maximum power density reaches 32 and 38 mW cm^−2^ for methanol and hydrogen fuel, respectively. The practical issue with SOFC while being fed by alcohol fuels is usually their low maximum power density (MPD) owing to higher anodic activation loss [41]. There are studies on alcohol-fueled SOFC that delivered very low MPD (<100 mW cm^−2^) even with noble metal-based anodes [42]. Furthermore, although Mo-based perovskites are commonly used as anode in SOFCs (due to their high redox stability and good carbon tolerance), their MPD is sometimes low even at 800 °C [43]. Similar La_0.5_Sr_0.5_Fe_0.9_Mo_0.1_O_3−d_ and Pr_0.6_Sr_0.4_Fe_0.7_Ni_0.2_Mo_0.1_O_3−δ_ delivered MPD of 190 mW cm^−2^ [44] and 162 mW cm^−2^ [45], respectively, at 650 °C in H_2_. In other studies, Sr_2_TiNi_0.5_Mo_0.5_O_6_ and La_0.3_Sr_0.7_Ti_0.97_Mo_0.03_O_3−d_ delivered MPD of ~100 mW cm^−2^ (at 700 °C) [27] and 60 mW cm^−2^ (at 800 °C) [24] in H_2_, respectively. Nevertheless, MPD is always affected not just by the anode composition, but also by TPB and microstructure of the anode and can be limited by OCV. Here, considering the low operating temperature of 600 °C and the dry methanol feed, besides the low achieved OCV, the cell delivered MPD of ~35 mW cm^−2^. The hypothesis is that either the LSCFMo composition or the electrode microstructure must be optimized to achieve faster electrocatalytic activity and higher power density. Still, LSCFMo is a promising anode material in SOFCs to process the oxidation of methanol with no carbon deposition. In cell Mo10, a similar behavior to Mo5 is observed at 600 °C (Figure 10c). The performances were improved by decreasing the methanol dilution, although the injection of He as carrier is necessary.

### 3.5. Carbon Deposition

Interestingly, after the tests, no trace of any carbon was found on the anode side nor in the anodic tube although all the fuels were dry. Any cracking is excluded from the shuttle mechanism which is a positive result and is accompanied by no carbon deposition at the end. However, there is a high possibility of Boudouard or water–gas reverse reactions occurrence at low temperatures, given, respectively, by

(6) $$2CO⟷{CO}_{2}+C{\Delta H}_{298}^{{}^{\circ}}=-171\;kJ/mol$$

(7) $$H_{2}+CO⟷H_{2}O+C{\Delta H}_{298}^{{}^{\circ}}=-131\;kJ/mol$$

Under reducing conditions, Mo^6+^ is easily reduced to Mo^5+^ which is accompanied by oxygen vacancy formation as

(8) $${2Mo}_{Mo}^{x}+O_{O}^{x}⟶{2Mo}_{Mo}^{.}+V_{O}^{..}+\frac{1}{2}O_{2}$$

Due to mixed-valent Mo^5+^/Mo^6+^, LSCFMo shows a good capability in fast loading/unloading oxygen ions, which provides the electrode with higher oxygen vacancies. As a result, TPB is enlarged in the anode and this leads to better electrocatalytic activity toward carbon oxidation. The cell integrity and mechanical strength are preserved during the tests.

## 4. Conclusions

Mo was doped successfully into LSCF by the auto-combustion method. An increased phase and microstructural stability were observed under reducing conditions by increasing the Mo dopant and substituting for Fe in LSCF. XRD analysis proved that LSCFMo10 retained the initial perovskite structure up to 60% under reducing conditions in H_2_ at 900 °C. Electrochemical measurements showed that biogas and methane are the least reactive fuels. Whereas LSCFMo is capable of oxidizing methanol through a “shuttle mechanism”. Since the first reaction is the dehydrogenation of methanol and it needs less activation energy with respect to methane direct oxidation, the perovskite follows this mechanism as proved by OCV and EIS. The very low series resistance proved a very good configuration of the realized cells. A 10% Mo dopant showed slightly better catalytic activity towards methanol oxidation than 5% Mo, and no trace of carbon deposition was found on the anode side after feeding the cells with dry biofuels. The chemical stability of LSCFMo is preserved after the electrochemical tests. Molybdenum’s redox gap overlapping with B-site cations, lower TEC, and quick surface exchange make it a suitable choice as a dopant in LSCF. As a result, the LSCFMo anode may be offered as an active electrocatalyst in LT-SOFCs for methanol oxidation.

## Data Availability

The original contributions presented in the study are included in the article, further inquiries can be directed to the corresponding author.
